# A methodological review of patient healthcare-seeking journeys from symptom onset to receipt of care

**DOI:** 10.1136/bmjgh-2024-016978

**Published:** 2025-05-16

**Authors:** Charity Oga-Omenka, Angelina Sassi, Nathaly Aguilera Vasquez, Namrata Rana, Mohammad Yasir Essar, Darryl Ku, Hanna Diploma, Lavanya Huria, Kiran Saqib, Rishav Das, Guy Stallworthy, Madhukar Pai

**Affiliations:** 1 School of Public Health Sciences, University of Waterloo, Waterloo, Canada; 2 McGill International TB Center, McGill University Health Centre, Montreal, Canada; 3 Epidemiology, Biostatistics, and Occupational Health, McGill University Faculty of Medicine and Health Sciences, Montreal, Canada; 4 McGill International Tuberculosis Centre, Montreal, Canada; 5 McGill University Department of Epidemiology Biostatistics and Occupational Health, Montreal, Canada; 6 McGill University International Tuberculosis Centre, Montreal, Canada; 7 McMaster University, Hamilton, Canada; 8 University of Waterloo, Waterloo, Canada; 9 University of Toronto, Toronto, Canada; 10 McGill University, Montreal, Canada; 11 Research Institute of the McGill University Health Centre, and McGill International TB Centre, Montreal, Canada, Research Institute of the McGill University Health Centre, Montreal, Canada; 12 Bill & Melinda Gates Foundation, Seattle, Washington, USA; 13 Epidemiology & Biostats, McGill University, Montreal, Canada

**Keywords:** Global Health, Qualitative study, Cross-sectional survey, Cohort study, Review

## Abstract

**Background:**

For many diseases, early diagnosis and treatment are more cost-effective, reduce community spread of infectious diseases and result in better patient outcomes. However, healthcare-seeking and diagnoses for several diseases are unnecessarily delayed. For example, in 2022, 3 million and 5.6 million people living with tuberculosis (TB) and HIV, respectively, were undiagnosed. Many patients never access appropriate testing, remain undiagnosed after testing or drop out shortly after treatment initiation. This underscores challenges in accessing healthcare for many individuals. Understanding healthcare-seeking obstacles can expose bottlenecks in healthcare delivery and promote equity of access. We aimed to synthesise methodologies used to portray healthcare-seeking trajectories and provide a conceptual framework for patient journey analyses.

**Design/methods:**

We conducted a literature search using keywords related to “patient/care healthcare-seeking/journey/pathway analysis” AND “TB” OR “infectious/pulmonary diseases” in PubMed, CINAHL, Web of Science and Global Health (OVID). From a preliminary scoping search and expert consultation, we developed a conceptual framework and honed the key data points necessary to understand patients’ healthcare-seeking journeys, which then served as our inclusion criteria for the subsequent expanded review. Retained papers included at least three of these data points.

**Results:**

Our conceptual framework included five data points and seven related indicators that contribute to understanding patients’ experiences during healthcare-seeking. We retained 66 studies that met our eligibility criteria. Most studies (56.3%) were in Central and Southeast Asia, explored TB healthcare-seeking experiences (76.6%), were quantitative (67.2%), used in-depth, semistructured or structured questionnaires for data collection (73.4%). Healthcare-seeking journeys were explored, measured and portrayed in different ways, with no consistency in included information.

**Conclusions:**

We synthesised various methodologies in exploring patient healthcare-seeking journeys and found crucial data points necessary to understand challenges patients encounter when interacting with health systems and offer insights to researchers and healthcare practitioners. Our framework proposes a standardised approach to patient journey research.

**WHAT IS ALREADY KNOWN ON THIS TOPIC**

- Accessing healthcare is challenging for half of the world’s population.
- Understanding healthcare-seeking obstacles can help to expose bottlenecks in healthcare delivery and improve access.

**WHAT THIS STUDY ADDS**

- We synthesised the different methodologies used by researchers to portray healthcare-seeking trajectories.
- We also provide a conceptual framework and recommendations for patient journey analyses.

**HOW THIS STUDY MIGHT AFFECT RESEARCH, PRACTICE OR POLICY**

- Our analysis revealed a lack of consistency in how patient journeys to care are represented and a notable complexity in generating insightful depictions of journeys to care.
- The use of our conceptual framework, namely the data points and indicators, could increase the reliability and generalisability of patient journey analyses.

## Introduction

 In 2021, 4.5 billion people or more than half of the world’s population lacked access to the healthcare they needed.^1^ Diagnoses for several diseases are unnecessarily delayed, contributing to prolonged suffering and avoidable deaths. For example, in 2022, an estimated 3 million and 5.6 million people living with tuberculosis (TB) and HIV, respectively, were undiagnosed.^2 3^ A significant number of individuals with life-threatening illnesses never access appropriate testing, remain undiagnosed after testing or drop out before or shortly after treatment initiation.^4^,^7^ Some reasons for these gaps in testing, diagnosis and treatment include individual and interpersonal dynamics like lack of information about the disease and available resources, stigma, financial and cultural factors, symptom minimisation, self-medication and mistrust of public sector healthcare^8^,^13^; as well as health system factors like poor coverage of services, low index of suspicion among providers, lengthy procedures, misdiagnosis and poor referral mechanisms between the public and private sectors.^1014^,^16^ These numbers underscore the substantial public health challenge that accessing healthcare poses for many individuals. Despite these barriers, early diagnosis and treatment remain critical for significantly improving clinical outcomes and reducing costs for both patients and the healthcare system, especially for infectious diseases like TB and HIV where early intervention is the most practical method to interrupt transmission.^10 17^

The WHO adopted the integrated people-centred health services framework in 2016 to prioritise people’s needs and expectations by engaging communities, reorienting models of care, coordinating services across sectors and strengthening governance and accountability.^18^,^20^ Person-centred care emphasises treating patients as individuals and focuses on providing integrated care, patient information and support and responding to patients’ values and preferences.^21 22^ To improve person-centred care, it is important to understand current patient journeys to care.^23^ These studies shed light on the obstacles patients encounter during healthcare-seeking, expose healthcare delivery bottlenecks and promote equitable access.^24^,^26^ Understanding patient journeys is critical for making services more patient focused, as healthcare-seeking involves multiple sequential decisions rather than single access points, allowing us to identify service delivery gaps and ensure early access to affordable, quality care.^1227^,^30^ While patient pathway analyses are emerging, lack of consistency in methods or shared terms and frameworks makes it difficult to interpret across settings. Our review aimed to address these inconsistencies by providing a synthesis of existing methodologies used to represent patients’ healthcare-seeking trajectories. Our framework aims to clarify existing methodologies and their applications in enhancing patient-centred care.

While also commonly referred to as patient pathways in literature, the healthcare-seeking journey analysis emphasised in this paper differs from the process analytics used to map and improve integration for in-hospital processes between the different departments^31^,^34^ or the pathway analysis to determine alignment between population health needs and available services.^35 36^ Patient journey analyses characterise and quantify the pathways to care of specific patients, detailing not only the total delays between key milestones^37^,^42^ but also the number and sequence of visits to healthcare providers.^27^,^2943 44^

Current literature lacks a comprehensive examination of the advantages and limitations of various methods for depicting healthcare-seeking trajectories and identifying the key variables that highlight these obstacles. Although the combination of conceptual and methodological reviews has been employed widely in the literature to critically appraise different research methods,^3045^,^47^ a focused review that synthesises methodologies specifically related to patient journey analyses remains limited. This review seeks to fill that gap by synthesising methodologies for analysing sequential healthcare-seeking trajectories between multiple providers, explicitly highlighting their advantages and limitations and providing a framework to complement existing research. While significant insights exist in the literature, our goal is to bring clarity to these methodologies, thereby improving understanding of patient journeys and their implications for patient-centred care. A conceptual review is focused on key data points or variables and their relationships, with the goal of categorising and describing them relevant to a particular topic.^48^,^50^ Methodological reviews differ from a traditional systematic review by prioritising methods over results,^51^,^54^ synthesising study quality by examining design, data collection and analysis.^51 52^ By synthesising these methodologies, this review aims to provide foundational insights for future research and interventions aimed at enhancing patient-centred care for TB and other diseases, emphasising the practical implications for healthcare delivery.

## Methods

### Review objectives

The primary aim of our review was to assess relevant current practices, methodologies and relevant data points used to map patient care trajectories for TB and related diseases, focusing on the patient healthcare-seeking journey from symptom onset to care outcome across diverse healthcare settings and conditions. The research question was: ‘How do the methodologies employed for patient journey analysis (PJA) differ, what are their strengths and limitations?’.

### Review approach

We use the conceptual and methodological review approaches, which adapt their scope and methods during the process.^52 54^ We used an iterative approach, using literature review and expert consultations to understand the patient healthcare-seeking journey. This entailed extensive database searches and consultations with experts in TB care and social science, and refining review findings based on iterative feedback.

### Search strategy and framework development

Our process comprised three steps: (1) A scoping literature search to identify relevant data points and methods; (2) expert consultation to identify conceptual papers and recommended measures, followed by the development of a conceptual map and (3) an updated literature search targeting papers that included identified data points.

#### Initial scoping literature search

We performed a scoping search on PubMed, Web of Science, Scopus and Google Scholar for publications on healthcare-seeking trajectories and delays related to TB diagnosis or treatment. The initial search was run in November 2021 (updated later) and was restricted to peer-reviewed publications in the English language with no date restrictions. We also consulted with TB care experts to identify additional papers.

#### Development of the conceptual framework

Our conceptual framework was developed through an initial literature review conducted by the lead author, an experienced public health researcher specialising in healthcare access and social determinants of health, and the graduate students within the research team, with inputs from the senior authors. The lead author reviewed the literature and identified multiple criteria—variables, factors and themes—that describe and influence patients’ journeys to care. The team engaged in several meeting discussions to refine and define the key features of the conceptual framework, ensuring that all relevant variables and factors were thoroughly considered. These meetings helped clarify the inclusion criteria and establish a cohesive draft. To further strengthen the framework, the lead author sought input from a diverse group of experts outside of the research team, including senior researchers, healthcare professionals and programme managers with extensive experience in global health as well as in TB and HIV care. This consultation process took place through a series of in-depth one-on-one meetings, workshops and a conference presentation, allowing for the identification of additional data points and ensuring that the framework reflected a comprehensive understanding of critical methodological considerations across varied contexts. As a result of this process, we decided to expand our initial literature search to cover methodologies used to describe patient healthcare-seeking journeys for infectious or pulmonary diseases, or diseases with non-specific symptoms or lengthy diagnostic procedures—including HIV, COVID-19, malaria and to a lesser extent, non-communicable diseases (NCDs). The papers selected for the conceptual framework were chosen based on key screening criteria to capture critical aspects of patient healthcare-seeking journeys. This included whether studies reported the number of provider encounters needed for diagnosis and treatment, the types of providers seen and the healthcare sector (public, private or informal). Additionally, studies were considered if they provided time increments for diagnosis and treatment, detailed the proportions of patients requiring specific numbers of encounters and described the facilities where diagnosis or treatment occurred. Some studies also analysed the alignment of services available to the population. These criteria ensured that the selected studies contributed meaningful insights to the framework. Defining the conceptual framework helped with the rest of the methodological review to identify the lack of consistency in methods and analytic approaches.

#### Updated literature search and inclusion criteria

We systematically searched PubMed, CINAHL, Web of Science, Global Health (OVID) and Scopus for publications on healthcare-seeking trajectories, delays to diagnosis or treatment, and access to diagnosis or treatment, for TB, HIV, malaria, COVID-19, and other respiratory and lung disorders. The initial search of November 2021 was updated in April 2022 and searched again in May 2023. Our search strategy can be found in online supplemental file 1.

To be included in our review, studies had to include at least three of the identified data points for assessing patient journeys within the healthcare system in low-income and middle-income countries (table 1). We excluded case studies of individuals; non-scientific publications such as opinions or editorials; systematic reviews and meta-analyses; studies on extrapulmonary TB, latent TB and paediatric TB (children under 15 years); studies focusing solely on the cascade of care; articles written in languages other than English or French; and articles that did not provide a full report of the results of an experimental study (abstract, reviews, commentaries, proposals, methodology papers or case study).

**Table 1:** The key data points of patient healthcare-seeking journey

Reference points	Data points	Purpose	How to measure (quantitatively and qualitatively)	Studies
Journey initiation	Date symptoms started	To determine the time when symptoms started or when patients recognised their symptoms	This is a date variable. Can also be expressed in time increments (eg, weeks, months).	2867 99
	Type of provider first visited after symptom onset	To identify the first healthcare provider that patients sought for their symptoms until diagnosis after recognition	For example, physician, nurse, pharmacist, laboratory technician, medicine vendor	2868 73 76 77 87 90 92
	Sector of facility first visited	To specify type of facility for that encounter	For example, private sector, public sector or informal sector	2868 73 76 87 92
Diagnostic process	Date of (or time to) diagnosis	To determine how long it took for patients to get diagnosed	This is a date variable. Can also be expressed in time increments (eg, weeks, months).	2867 99 101 104
	Number of provider encounters to diagnosis	To identify the number of visits it took for patients to go from the first encounter until diagnosis	1st encounter, 2nd encounter, etc.	2843 67 72 75 77 79
	Type of provider for each encounter to diagnosis	To specify the type of provider the patients saw at each encounter	For example, physician, nurse, pharmacist, laboratory technician, medicine vendor	2868 71 73 76 77 87 90 92
	Sector of facility for each encounter	To specify type of facility for where patients were diagnosed	For example, private sector, public sector or informal sector	2868 71 73 76 87 92
	Proportions of patients per number of encounters to diagnosis	To determine the proportion of patients who had that many encounters. Also helps to identify missed opportunities for that provider	Expressed as % of 1st encounter, 2nd encounter, etc; or number of patients at each reference point, for example, ‘20% of patients had 1–2 encounters, 30% had 3 encounters…’	^28 68 70 71 74 81 85 87 91 95 96 99 100 103 106 110 111 116 118 120^
Treatment process	Date of (or time to) treatment initiation	To determine how long it took for patients to start treatment once diagnosed	This is a date variable. Can also be expressed in time increments (eg, weeks, months).	2868 74 76
	Number of provider encounters to treatment initiation	To identify the number of visits to a provider that it took for patients to start treatment after diagnosis.	1st encounter, 2nd encounter, etc.	6871 80 83 85 89 93 95 101 102 104 107 109 111 113 118 122 123 125
	Type of provider for each encounter to treatment initiation	To specify the type of provider the patients saw at each encounter	For example, physician, nurse, pharmacist, laboratory technician, medicine vendor	68 7193 95 96 98 99 101 102 104 107 109 111 113 119 121 127
	Sector of facility for each encounter to treatment initiation	To specify type of facility where patients were started on treatment	For example, private sector, public sector or informal sector	6871 93 95 96 98 99 101 102 104 107 109 111 113 127 129
	Proportions of patients per number of encounters to treatment initiation	To determine the proportion of patients who had that many encounters. Also helps to identify missed opportunities for that provider	Expressed as % of 1st encounter, 2nd encounter, etc; or number of patients at each reference point, for example, ‘20% of patients had 1–2 encounters, 30% had 3 encounters…’	^70 71 84 91 93 95 101 103 113 118 120 128^

References retrieved from all databases were imported into Covidence. Title and abstract screening were carried out by five members of the team (NAV, CO-O, DK, AS and MYE) and full-text screening by four members of the team (CO-O, LH, NAV and MYE), meeting regularly to review progress and resolve conflicts. Agreement on inclusion between two reviewers was required for inclusion into the study. Conflicts were resolved by a third reviewer when necessary.

There are no clear guidelines for appraising the quality and risk of bias for methodological reviews.^51 52^ The focus of the appraisal process for this type of review should be on distinguishing between papers stemming from flawed empirical studies and those presenting well-argued theories.^51^ We used the JBI Critical Appraisal Checklists^55^ for each applicable study type (qualitative, cross-sectional, cohort or prevalence study) and the Mixed Methods Appraisal Tool^56^ for mixed-methods studies to evaluate the methodological rigour and reliability of the included studies and identify potential biases and limitations within the studies (online supplemental file 2). Quality assessment was carried out by six members of the team (AS, NAV, RD, DK, MYE and KS) using an electronic form which was piloted by AS and NR (online supplemental file 3). Quality assessment for each paper was done by two independent reviewers, and answers were finalised during a consensus meeting led by AS and MYE.

### Data extraction

Data extraction was done by six members of the team (AS, MYE, NR, DK, HD and CO-O) using an electronic data extraction form hosted on Covidence (online supplemental file 4). The data extraction form was piloted by CO-O, NAV, LH and NR prior to data extraction. The data extraction form aimed to collect general information (author name, publication year, country/setting, disease type, study design/data sources, study type and sample size), the journey criteria included for diagnosis and treatment, a general description of the methodology and analysis for each study, as well as strengths and limitations. Data extraction for each paper was done by two independent reviewers, and answers were finalised during a consensus meeting led by AS and MYE.

### Patient and public involvement

We did not engage patients or the public as this is a review of completed studies.

## Results

### Definition of key data points and the conceptual map

The care continuum describes the sequence of activities needed to be fully engaged in clinical care for diseases like TB and HIV that require lengthy health system procedures to achieve an outcome.^57^,^59^ Our patient journey framework, developed after the initial review of literature, highlights the patient healthcare-seeking trajectory across three reference points: symptom recognition, diagnosis and treatment initiation. Our review illustrates how patients consult various types of providers in differing numbers as they seek care between these reference points. To characterise each visit within a patient’s journey, we identified five key data points: (1) the date of the first visit relative to symptom onset, (2) the type of provider or facility visited (eg, chemist, general practitioner, specialist, laboratory, hospital), (3) the sector of the provider or facility (for-profit, non-profit, public), (4) the sequence of the visit (first visit, second visit, etc) and (5) the outcome of the visit (no outcome, diagnosis and treatment initiation). Using these data points, we can compute various indicators for both individual patients and aggregated patient populations. These measures include (1) the number of days and (2) visits until care outcome (diagnosis or treatment initiation), (3) the proportion of visits to different types of providers, (4) the contribution of various provider types to care outcome (diagnosis or treatment) as well as to (5) delays or missed opportunities and (6) the number of patients exhibiting different patterns or sequences of provider visits. This comprehensive analysis can inform strategies to optimise patient care experiences and reduce delays in diagnosis and treatment. While the framework presents these stages in a linear format, it is essential to clarify that this structure does not overlook the inherently complex and often nonlinear nature of healthcare-seeking behaviour. Instead, it serves as a foundational guide to facilitate understanding of patient pathways amidst the messiness of real-world experiences.

**Figure 1:**
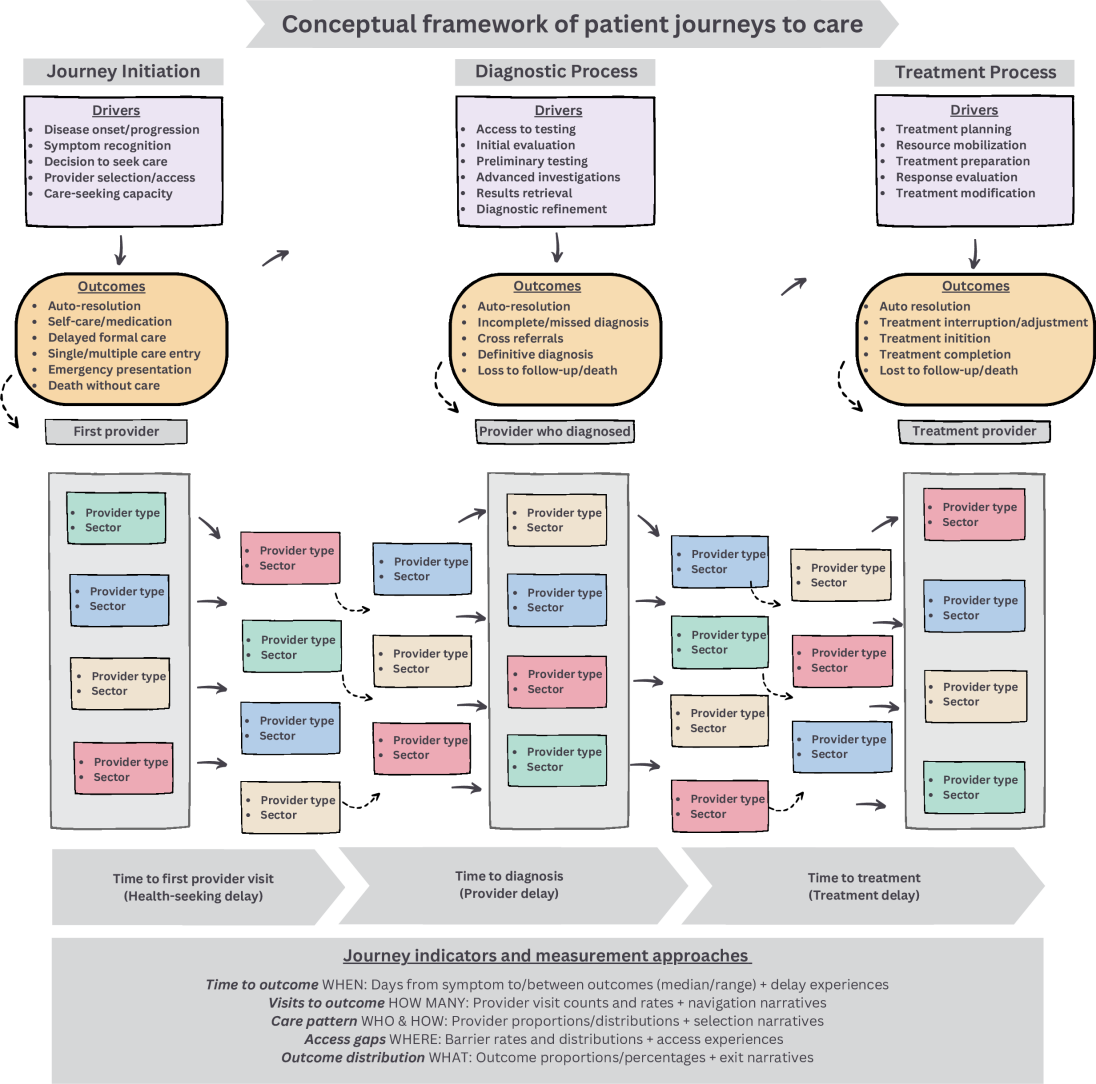
Conceptual framework of healthcare-seeking journeys.

Our conceptual framework (figure 1) illustrates the steps in a patient journey and the time delays between key milestones of symptom recognition, diagnosis and treatment. Starting with symptom recognition, factors like symptom minimisation and lack of knowledge of services have been shown to influence healthcare-seeking delays, while the type and sector of first provider have been shown to influence the number of subsequent encounters before and timeliness of diagnosis.^10 11 60 61^ Several health system factors have been shown to influence the timing and number of encounters between the reference points of diagnosis and treatment.^10 11 28^ Delays are conceptualised as healthcare-seeking, provider and treatment delays, with diagnostic delays being the sum of healthcare-seeking and provider delays, and health system delays representing the sum of provider and treatment delays.^27 29^ We acknowledge that the healthcare-seeking process is often complex and non-linear, with family members or proxies frequently influencing the patient’s journey, such as when a family member shares medications with their family members. Although these nuances are not explicitly captured within the framework, our model aims to streamline and clarify such complexities by focusing on the key stages and decision points in the care-seeking pathway. This approach helps to pinpoint critical areas for intervention, even as it reflects the reality that patient experiences do not always follow a straightforward, linear trajectory.^62^ Furthermore, while our framework is particularly relevant for TB and HIV, where healthcare-seeking challenges are often similar, it has the flexibility to be adapted for more intricate pathways, such as those involved in the care for NCD like cancer, where diagnosis is progressive, and treatment involves multiple modalities. This adaptability ensures the framework’s applicability across a range of conditions, while retaining a strong focus on TB and HIV.

### Characteristics of included studies

**Figure 2:**
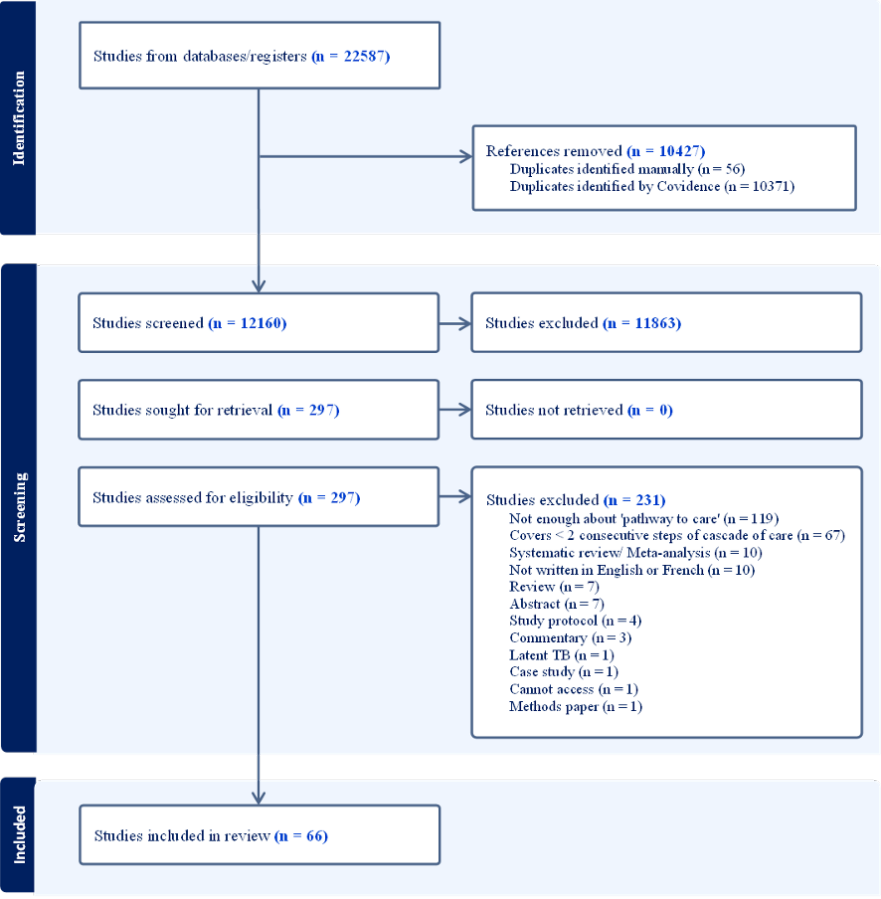
PRISMA (Preferred Reporting Items for Systematic reviews and Meta-Analyses) diagram of study selection process.

The PRISMA (Preferred Reporting Items for Systematic reviews and Meta-Analyses) flow diagram (figure 2) shows search results from the five main databases. Out of the 12 160 studies whose titles and abstracts were initially screened from those databases, 297 studies were assessed for retrieval and eligibility, 231 were excluded through full-text review and 66 studies were retained that met our eligibility criteria. Each of these 66 studies (our unit of analysis) was a patient journey analysis publication.

Out of the 66 retained studies, we assessed 21 to be of high quality, 37 to be medium quality and 8 to be of low quality. There were no major differences in the methods for the studies assessed with different qualities.

Table 2 summarises journey methodology characteristics of included studies. Many studies were quantitative (82%), were conducted in lower-middle-income countries (56%), in Asia (59%), and covered TB care journeys (67%). Other diseases include the following: cancer (6.1%), acute febrile illnesses (4.5%), malaria (4.5%), chest symptomatic (3.0%), multiple infectious diseases (3.0%), NCDs (3.0%), sexually transmitted infections (3.0%), under-5 mortality (3.0%) and COVID-19 (1.5%). The study design most often used was cross-sectional study (77%) and the most common quality level was medium (56%).

**Table 2:** Characteristics of included manuscripts

Characteristic	N=66*
**Year of publication**	2017 (1997–2022)
Region	
Asia (Bangladesh, China, India, Indonesia, Nepal, Singapore, Syrian Arab Republic, Taiwan, Tajikistan, Thailand)	39 (59%)
Africa (Burkina Faso, Ethiopia, Gambia, Kenya, Malawi, Mozambique, Nigeria, Senegal, South Africa, Uganda, Zimbabwe)	19 (29%)
Oceania (Australia, Vanuatu)	3 (4.5%)
Americas (Brazil, Canada)	2 (3.0%)
Europe (Switzerland, UK)	2 (3.0%)
Multicountry (France, Germany, Japan, USA)	1 (1.5%)
Income level	
Low income	10 (15%)
Lower middle income	37 (56%)
Upper middle income	11 (17%)
High income	8 (12%)
Low to middle income (multicountry)	0 (0%)
Disease	
Tuberculosis	44 (67%)
Cancer	4 (6.1%)
Acute febrile illnesses	3 (4.5%)
Malaria	3 (4.5%)
Non-communicable diseases	3 (4.5%)
Chest symptomatic	2 (3.0%)
Multiple infectious diseases	2 (3.0%)
Sexually transmitted infections	2 (3.0%)
Under-5 mortality	2 (3.0%)
COVID-19	1 (1.5%)
Study design	
Cross-sectional study	51 (77%)
Qualitative Research	7 (11%)
Mixed methods	5 (7.6%)
Cohort study	3 (4.6%)
Data type	
Quantitative	54 (82%)
Qualitative	7 (11%)
Mixed	5 (7.6%)
Quality appraisal	
Low quality	8 (12%)
Medium quality	37 (56%)
High quality	21 (32%)
Total sample size	283 (26–23 961)
Number of male participants†‡	186 (2–12 987)
Number of female participants†§	100 (12–10 974)

* Median (range); n (%).

† Sex of participants unspecified (n=4).

‡ Only women included (n=1).

§ Only men included (n=1).

**Figure 3:**
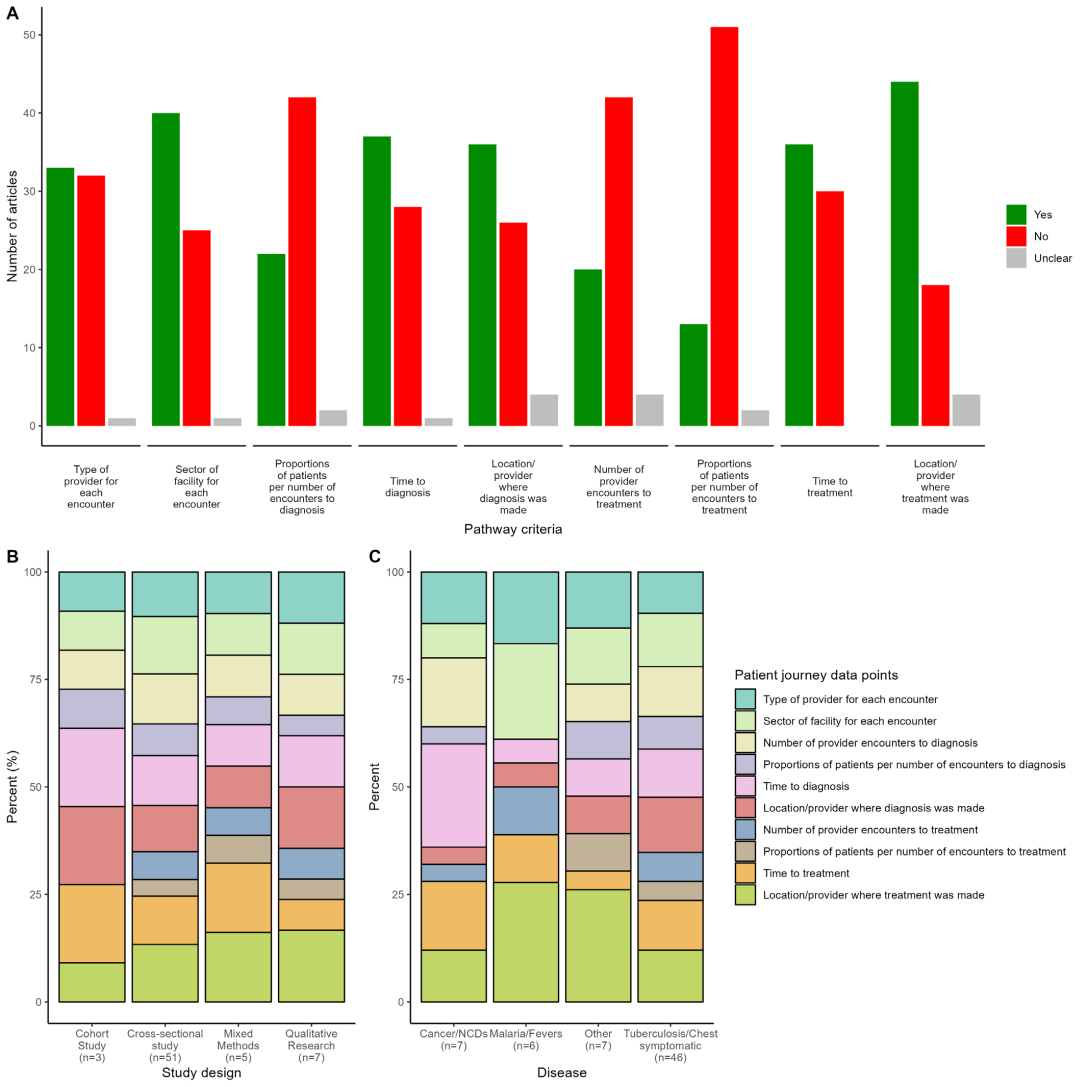
Distribution of journey data points across all included studies, by disease types and study designs. (A) Distribution of patient healthcare-seeking journey data points. (B) Coverage of patient journey data points by disease studied. (C) Coverage of patient journey data points by study design.

Figure 3A summarises the distribution of patient healthcare-seeking data points aggregated across all included studies. The majority included information on location/provider where treatment was initiated (67%), sector of the providers visited (61%), time to diagnosis (56%), location/provider where a diagnosis was made (55%), the number of provider encounters to diagnosis (53%) and type of the providers visited (50%). Proportions of patients per number of encounters to diagnosis (33%) and treatment (20%), and number of provider encounters to treatment (30%) were less commonly explored in the included papers.

Figure 3B illustrates the distribution of patient healthcare-seeking data points across four disease groups: cancer/NCDs, malaria/fever, other and TB/chest symptoms, respectively. Notably, while most patient healthcare-seeking data points were reported within studies spanning these disease groups, the distributions were variable. For instance, the proportion of patients per number of encounters to treatment was not found in studies addressing cancer/NCDs. In contrast, research on malaria/fever more frequently reported the number of provider encounters to treatment but did not include the number of provider encounters to diagnosis, proportion of patients per number of encounters to diagnosis and proportion of patients per number of encounters to treatment. Additionally, studies examining other diseases omitted the number of provider encounters to treatment. Studies focused on TB reported all key data points, notably documenting the sector of facility for each encounter at the highest rate.

Figure 3C again depicts the coverage of patient journey data points across various study designs: cohort study, cross-sectional study, mixed-methods study and qualitative study. While most categories of data were present across all study designs, notable differences in distributions emerged. Cohort studies reported the highest proportions of patients per number of encounters to diagnosis but did not provide data on the number of provider encounters to treatment. Conversely, cross-sectional studies typically reported the lowest figures across all key data points, whereas mixed-methods studies consistently yielded the highest averages for these points. Notably, studies using three of the four study design types—cross-sectional, mixed methods and qualitative research—successfully reported all key data points.

Table 3 details the depictions of healthcare-seeking journeys and statistical methods used in the included studies. The most common forms of depicting patient healthcare-seeking journeys were tables quantifying delays and/or journeys (48%) and flow charts (48%). Descriptive statistics (88%), multivariate regressions (45%), χ^2^ test (45%) and logistic regression (36%) were the most frequently used statistical methods.

**Table 3:** Journey depictions and statistical methodologies

Characteristic	N=66
**Depictions of patient journeys to care**	
Tables quantifying delays/journeys	32 (48%)
Flow chart	32 (48%)
Bar chart	12 (18%)
Authors’ own depiction (other)	9 (14%)
Matrix representing patient journeys	6 (9.1%)
Diagram summarising types of delays	5 (7.6%)
Sankey chart	3 (4.5%)
Scatter plot	1 (1.5%)
**Statistical methodologies used**	
Main statistical method used per article
Logistic regression only	18 (27%)
χ2 test only	7 (11%)
Independent t-test and χ2 test only	7 (11%)
More than one regression method	7 (11%)
Thematic analysis only	7 (11%)
Descriptive statistics only	5 (7.6%)
Independent t-test only	5 (7.6%)
Mixed methods	4 (6.1%)
Linear regression only	3 (4.5%)
Analysis of variance (ANOVA) test only	1 (1.5%)
Quantile regression only	1 (1.5%)
Survival analysis only	1 (1.5%)
Descriptive statistics	58 (88%)
χ2 test	30 (45%)
Independent t-test	22 (33%)
Fisher’s exact test	8 (12%)
Mann-Whitney test	8 (12%)
Kruskal-Wallis test	4 (6.1%)
ANOVA	3 (4.5%)
Multivariate regression (all types)	30 (45%)
Logistic regression	24 (36%)
Linear regression	8 (12%)
Survival analysis	3 (4.5%)
Poisson regression	2 (3.0%)
Quantile (median) regression	2 (3.0%)
Thematic analysis of qualitative data	11 (17%)

Table 4 depicts the outcome measures and independent variables used in the included articles. Overall, patient delay (58%) was the most common outcome measure for the 66 included studies. Over one-third (42%) of included articles did not report all possible delays that could have been reported in the manuscript; for example, if the authors reported diagnostic delay (defined^27 29^ as the number of days between the onset of symptoms and the date of diagnosis and composed of patient delay and provider delay) but did not also report both patient and provider delay. Other than overall delay, the most reported outcome measures of patient journeys were number of visits to diagnosis (36%), sector of facility and type of provider for all instances of healthcare-seeking (36% and 33%, respectively), and type of provider for initial healthcare-seeking visit (32%). Time to initial healthcare-seeking was reported in 29% of included studies. Patient age (97%) and sex or gender (92%) were the two most used independent variables among the 66 included studies, followed by level of education (62%) and occupation (59%). Among patient health status and patient behaviour variables, type of disease (30%), presence of any comorbidities (27%) and HIV status (27%) were often included as independent variables. Some patient healthcare-seeking journey characteristics were used as independent variables in analyses of delays, for example, the number of visits (30%), sector of facility (39%) and type of provider (38%) of the initial healthcare-seeking visit.

**Table 4:** Outcome measures and independent variables used

Type	Variable	N=66*
**Outcome measures**	**Delays and time measures**	
	Healthcare-seeking or patient delay or time to initial healthcare-seeking	38 (58%)
	Provider delay	15 (23%)
	Treatment delay or time to treatment	16 (24%)
	Diagnostic delay or time to diagnosis	18 (27%)
	Health system delay	14 (21%)
	Total delay	18 (27%)
	Manuscript did not report all possible delay components	28 (42%)
	Manuscript reported ‘durations’ and defined ‘delays’ as durations above a given threshold	11 (17%)
	**Patient journey data points**	
	Number of visits to diagnosis	24 (36%)
	Number of visits to treatment initiation	12 (18%)
	Type of provider for all instances of healthcare-seeking	22 (33%)
	Sector of facility for all instances of healthcare-seeking	24 (36%)
	Type of provider for initial healthcare-seeking visit	21 (32%)
	Sector of facility for initial healthcare-seeking visit	15 (23%)
	Type of provider for place of diagnosis	5 (7.6%)
	Sector of facility for place of diagnosis	8 (12%)
	Type of provider for place of treatment	4 (6.1%)
	Sector of facility for place of treatment	6 (9.1%)
	Patient sought care for their symptoms	3 (4.5%)
	**Date and time to event measures**	
	Date of symptom onset	5 (7.6%)
	Date of initial healthcare-seeking	6 (9.1%)
	Date of diagnosis	5 (7.6%)
	Date of referral for treatment	3 (4.5%)
	Date of treatment initiation	3 (4.5%)
	Date of treatment completion	2 (3.0%)
	Survival analysis (time to event/death)	2 (3.0%)
	**Others**	
	Costs of care (at any point)	11 (17%)
	Reasons for seeking care	5 (7.6%)
	Patient knowledge of disease	2 (3.0%)
	Qualitative study	4 (6.1%)
**Independent variables**	**Demographic characteristics**	
	Age	64 (97%)
	Sex/gender	61 (92%)
	Level of education	41 (62%)
	Occupation	39 (59%)
	Income level	30 (45%)
	Literacy	21 (32%)
	Urban/rural	19 (29%)
	Marital status	18 (27%)
	Distance from nearest health facility	13 (20%)
	Place of residence	11 (17%)
	Household size	11 (17%)
	Socioeconomic status	10 (15%)
	Race/ethnicity	6 (9.1%)
	Health insurance status	5 (7.6%)
	Family structure	5 (7.6%)
	Religious status	4 (6.1%)
	Type of house	4 (6.1%)
	**Patient health status/behaviour**	
	Type of illness	20 (30%)
	Comorbidities	18 (27%)
	HIV status	18 (27%)
	Knowledge of disease	16 (24%)
	Symptoms experienced	15 (23%)
	Diabetes	15 (23%)
	Smoking status	14 (21%)
	Alcohol use	11 (17%)
	History of TB	9 (14%)
	Severity of illness	8 (12%)
	Type of TB (sputum smear positive/negative)	7 (11%)
	Heart disease, cardiovascular disease or hypertension	7 (11%)
	Duration of symptoms	5 (7.6%)
	Lung disease	4 (6.1%)
	Asthma	4 (6.1%)
	Stigma experienced	4 (6.1%)
	Height/weight/BMI	4 (6.1%)
	Contact with person with TB	3 (4.5%)

* n (%)

BMI, body mass index; TB, tuberculosis.

## Discussions

In our review, we aimed to understand and characterise methodologies used to assess patient journeys to care. Understanding patient journeys helps pinpoint bottlenecks and interventions to minimise missed diagnostic and treatment opportunities among various types of healthcare providers, improve hospital coordination and reduce overall delays in care. Moreover, accurately characterising aggregate patient journeys between different locations or across different times enhances progress monitoring and policymaking. This requires developing better, consistent metrics and more insightful data visualisations.

However, patient journeys rarely follow linear trajectories through formal healthcare sectors. Our review revealed complex, often circular patterns involving both healthcare providers and informal caregivers. Our analyses track the number, type and sector (private, public or informal) of healthcare provider visits, offering a framework to capture key elements of these non-linear patient journeys. For example, as shown in numerous other studies, patients may simultaneously engage multiple care sources, rely on family members as healthcare proxies (eg, obtaining medicines from pharmacists) or seek care entirely outside formal facilities.^41 42 44 62 63^ These nuanced dynamics challenge traditional journey models and demand more sophisticated analytical approaches.

Our analysis also revealed a lack of consistency in how patient journeys to care are represented and a notable complexity in generating insightful depictions of journeys to care, indicating the challenges in using data to guide healthcare interventions effectively. The scarcity of tools and clear guidelines for analysing and presenting such complex data further complicates this task, highlighting an urgent need for simple, open-access tools and standardised analytical methods to improve our understanding of patient healthcare-seeking journeys.

We posit that our proposed conceptual framework, identified data points and indicators address this knowledge gap, offering some guidance to improve consistency in the analysis of patient journey or healthcare-seeking experiences.

The reviewed papers typically addressed an average of four identified key data points, with patient delays and the type or sector of facility where the patient was diagnosed or treated being the most often examined. We also noted that the number of provider visits needed for diagnosis or treatment was only reported in 24% and 12%, respectively. In our view, the number of visits to diagnosis and treatment is among the most important data points for understanding patient care barriers, as both give us an insight into delays as well as the direct and indirect costs of care. Overall, as none of these factors alone fully characterise patient experiences, or highlight missed diagnostic and treatment opportunities, reporting all data points relevant to the outcome under review (diagnosis or treatment) is crucial for a comprehensive view of patient care journeys. Thus, our methodological review highlighted the key features of patient trajectories (table 1) that offer insights into the missed opportunities. Additionally, we recommend additional statistical analyses to identify risk factors for having more provider visits or longer delays in the patient care continuum using patients’ sociodemographic characteristics and health status (table 4). We propose the need for an open-source analysis package for Patient Journey Analyses.

### Strengths and limitations of study designs

We assessed the strengths and limitations for each study design to better understand their contributions to our findings on patient journeys to care. Cross-sectional studies provide an overview of patient proportions and care-seeking behaviours and quantify patient proportions between visits or delays. However, the variability in tools and measures across studies complicates consistent aggregation of results, leading to potential inconsistencies in our findings. For instance, diverse assessment methods—varying from structured questionnaires to retrospective chart reviews and prospective data collection techniques—complicate the quantification and understanding of patient journeys, as inconsistent definitions of key concepts like ‘delay’ hinder comparisons and obscure insights, emphasising the need for standardised metrics to improve the reliability of future research and identify systemic barriers to care. Cohort studies excel in examining care-seeking behaviours over time and identifying risk factors for delays, providing insights into temporal relationships and complex journeys. Despite their strengths, these studies often require large sample sizes and are vulnerable to recall bias, particularly in low-prevalence diseases. This limitation may affect the reliability of findings regarding the timing and number of provider visits that contribute to delays in diagnosis and treatment. Despite being more resource-intensive, mixed-methods research combines quantitative data with rich qualitative insights from key informants. While this approach provides a comprehensive view of obstacles faced in care-seeking, it can be resource-intensive and may result in smaller sample sizes, limiting the generalisability of findings. Similarly, qualitative research offers deep insights into the patient journey, allowing for flexibility in adapting to various contexts, but also faces limitations related to recall bias, high costs and small sample sizes, which can ultimately skew the understanding of patient experiences.

### Implications for future research

Patient journey analyses are prone to potential inconsistencies in data collection, particularly regarding sampling of patients, measuring encounters by number of providers or visits and the measurement of time reference points (symptom onset, diagnosis and treatment). This can also be seen by the fact that two-thirds of the articles included in this review were assessed as low or medium quality. Future research should focus on developing clear, practical recommendations for conducting patient journey analyses and creating open-access tools to overcome methodological and resource limitations. These advancements are crucial for researchers and teams aiming to analyse patient journeys effectively to identify and address care bottlenecks. Learning from our experiences and adopting these strategies will improve the quality, reliability and comparability of patient journey analyses in future studies.

To mitigate recall bias, researchers need to pilot test study instruments, train data collection teams, employ specific sampling strategies (eg, recruiting patients diagnosed within the last 6 months) and use data triangulation, including mixed methods and comparison to earlier studies where possible. Additionally, data collectors need to be creative in helping patients recall key dates, for example, with use of local or national events, and/or use clinical records to verify patient-reported dates, where feasible. Furthermore, integrating mobile applications that enable patients to document their experiences at critical time points can improve the accuracy and timeliness of data collection. Researchers should also explore linking various data sources, including routinely collected health service data and cost/billing information, to provide a more comprehensive understanding of patient experiences and outcomes.

Conceptually, incorporating patient cost surveys into journey analyses is another opportunity for a comprehensive understanding of patient care barriers, particularly where these include incurred costs for all healthcare provider visits. Cost surveys are often conducted by economists with a primary focus on cost analysis and rarely provide detailed insights into the nuances of patient journeys. Conversely, patient journey analyses rarely include cost data. This disconnect between cost analysis and journey mapping presents a significant gap in the literature, and integrating both aspects could provide a more holistic view of patient experiences and economic impacts. To address this gap, we recommend that future patient cost surveys be designed to enable rigorous analysis of patient journeys, and vice versa. Such an approach would facilitate a better understanding of both the financial and experiential data points of patient care-seeking behaviours. Ultimately, this dual-focus approach could significantly enhance the quality of insights derived from patient surveys, leading to improved patient-centric service delivery and better-informed healthcare policy decisions.

### Limitations

This methodology review has some important limitations. First, as noted in our results, the framework presents stages of patient journeys to care in a linear format, which can oversimplify the complex and often non-linear nature of healthcare-seeking behaviour. We hope this framework serves as a foundational guide to facilitate measurement and understanding of patient pathways amidst the messiness of real-world experiences.

Second, the research team was composed of public health and global health researchers and practitioners and lacked background knowledge on other fields that may have insights to contribute to this work. Our reviewers noted that patient journeys in the field of anthropology are sometimes denoted by the term ‘therapeutic itineraries’,^64^,^66^ which was not included in our search criteria.

### Conclusions

Our findings underscore the critical need for better approaches to compare patient journeys and develop interventions, emphasising the importance of clear guidelines and accessible tools to facilitate this analysis. This work lays the groundwork for future efforts to enhance access, quality and equity in healthcare service provision.

## Supplementary material

10.1136/bmjgh-2024-016978 online supplemental file 1

10.1136/bmjgh-2024-016978 online supplemental file 2

10.1136/bmjgh-2024-016978 online supplemental file 3

10.1136/bmjgh-2024-016978 online supplemental file 4

## Data Availability

All data relevant to the study are included in the article or uploaded as supplementary information.
