# Supplementary material for: A methodological review of patient healthcare-seeking journeys from symptom onset to receipt of care
Source: BMJ Glob Health. 2025 May 16;10(5):e016978. doi: 10.1136/bmjgh-2024-016978 (PMC12086929; doi:10.1136/bmjgh-2024-016978)
Supplement: online supplemental file 1 [file bmjgh-10-5-s001.pdf]

Supplement 1 – Search strategy

CINAHL

Data points	Key words
Patient journey	(TI ("Patient pathway analys*" OR PPA OR "treatment delay" OR "health-seeking" OR “healthcare-seeking” "health-seeking delay" OR "care seeking" OR "cascade of care" OR "access to care" OR "healthcare delivery" OR "diagnostic delay" OR "care seeking delay" OR "barriers and facilitators to care" OR "missed opportunit*" OR “care pathway*”) OR AB ("Patient pathway analys*" OR PPA OR "treatment delay" OR "health-seeking" OR "health-seeking delay" OR "care seeking" OR "cascade of care" OR "access to care" OR "healthcare delivery" OR "diagnostic delay" OR "care seeking delay" OR "barriers and facilitators to care" OR "missed opportunit*" OR “care pathway*”) OR (MH "Diagnosis, Delayed"))
Tuberculosis OR other pulmonary diseases OR selected infectious diseases	((MH "Tuberculosis, Pulmonary") OR TI (tb OR tuberculosis) OR AB(tb OR tuberculosis) OR (MH "Mycobacterium Tuberculosis")) OR (MH "respiratory tract diseases") OR (MH “respiratory disease) OR (MH “respiratory infection”) OR (MH "malaria") OR TI (malaria OR HIV OR covid) OR AB (malaria OR HIV OR covid) OR (MH “human immunodeficiency virus") OR (MH "sars-cov-2") OR (MH "covid-19")

PubMed

Data points	Key words
Patient journey analysis	“Patient pathway analys*” [tw] OR PPA [tw] OR “treatment delay” [tw] OR “healthcare-seeking” [tw] OR “health-seeking delay” [tw] OR “care seeking” [tw] OR “cascade of care” [tw] OR “access to care” [tw] OR “healthcare delivery” [tw] OR “diagnostic delay” [tw] OR “pathway analysis” [tw] OR “care seeking delay” [tw] OR “barriers and facilitators to care” [tw] OR “missed opportunit*” [tw] OR “care pathway*” [tw] OR "delayed diagnosis" [MeSH] OR "critical pathways" [MeSH] OR "patient acceptance of health care"[MeSH]
Tuberculosis	Tuberculosis, Pulmonary [Mesh] OR tb[tw] OR tuberculosis[tw] OR Mycobacterium tuberculosis[Mesh]
Other pulmonary diseases and	"respiratory tract diseases"[MeSH Terms] OR "respiration disorders"[MeSH Terms] OR respiratory disease[Text Word] OR “respiratory infection” OR "malaria"[MeSH Terms] OR malaria [tw]

relevant infectious diseases	OR "hiv"[MeSH Terms] OR HIV[tw] OR “human immunodeficiency virus" [tw] "sars-cov-2"[MeSH Terms] OR "covid-19"[MeSH Terms] OR covid [tw]

Web of Science

**Data points**	**Key words**
Patient journry analysis	TS=("Patient pathway analys*" OR PPA OR "treatment delay" OR "healthcare-seeking" OR "health-seeking delay" OR "care seeking" OR "cascade of care" OR "access to care" OR "healthcare delivery" OR "diagnostic delay" OR "care seeking delay" OR "barriers and facilitators to care" OR "missed opportunit*" OR “care pathway*”)
Tuberculosis	TS=(tb OR tuberculosis)
Other pulmonary diseases and relevant infectious diseases	TS=("respiratory tract diseases" OR "respiration disorders" OR “respiratory disease” OR “respiratory infection” OR "malaria" OR OR HIV OR “human immunodeficiency virus" OR "sars-cov-2" OR "covid-19" OR covid)

Global Health via Ovid

**Data points**	**Key words**
Patient journey analysis	("Patient pathway analys*" OR PPA OR "treatment delay" OR "healthcare-seeking" OR "health-seeking delay" OR "care seeking" OR "cascade of care" OR "access to care" OR "healthcare delivery" OR "diagnostic delay" OR "care seeking delay" OR "barriers and facilitators to care" OR "missed opportunit*" OR “care pathway*”).mp.
Tuberculosis	(tb OR tuberculosis).mp.
Other pulmonary diseases and relevant infectious diseases	("respiratory tract diseases" or "respiration disorders" or "respiratory disease" or "respiratory infection" or malaria or HIV or "human immunodeficiency virus" or "sars-cov-2" or covid-19 or covid).mp.
