# Supplementary material for: A methodological review of patient healthcare-seeking journeys from symptom onset to receipt of care
Source: BMJ Glob Health. 2025 May 16;10(5):e016978. doi: 10.1136/bmjgh-2024-016978 (PMC12086929; doi:10.1136/bmjgh-2024-016978)
Supplement: online supplemental file 2 [file bmjgh-10-5-s002.pdf]

## *Supplement 2 – Data extraction form*

### General information

| | |
|---|---|
| First author last name | [Open text field] |
| Year of publication | [Open text field] |
| Country/setting<br>as specific as possible | [Open text field] |
| Disease type<br>TB, HIV, malaria, etc. | [Open text field] |
| Study design/Data sources<br>e.g. population-based surveys, patient interviews | [Open text field] |
| Quantitative/Qualitative/Mixed | [Open text field] |
| Total sample size<br>Indicate sex of participants if available | [Open text field] |

### Patient healthcare-seeking journey data points included

| | |
|---|---|
| <u>Number of provider encounters to diagnosis</u><br>Does the paper report the number of visits to a HC provider that it took for patients to go from initial healthcare-seeking for their symptoms until diagnosis? e.g. "On average, patients had 5 encounters before being diagnosed" | ○ Yes<br>○ No<br>○ Unclear |
| <u>Type of provider for each encounter</u><br>Does the paper mention which type of provider the patients saw? (expressed as % of 1st encounter, 2nd encounter, etc.; or, number of patients who went to each type of provider) | ○ Yes<br>○ No<br>○ Unclear |
| <u>Sector of facility for each encounter</u><br>Does the paper mention whether the providers are in the private sector, public sector, or informal sector? | ○ Yes<br>○ No<br>○ Unclear |
| <u>Proportions of patients per number of encounters to diagnosis</u><br>For each number of encounters, do they say the proportion of patients who had that many encounters between initial healthcare-seeking and diagnosis? e.g. "20% of patients had 1-2 encounters, 30% had 3 encounters…" | ○ Yes<br>○ No<br>○ Unclear |
| <u>Time to diagnosis</u><br>Do they give how long it took for patients to get diagnosed in time increments (e.g. precise dates or time in days, weeks, months, etc.)? | ○ Yes<br>○ No<br>○ Unclear |
| <u>Location/provider where diagnosis was made</u><br>Do they say what type of health facility or provider the patients received their diagnosis from? | ○ Yes<br>○ No<br>○ Unclear |
| <u>Number of provider encounters to treatment</u><br>Does the paper report the number of visits to a HC provider that it took for patients to start treatment from the first provider encounter | ○ Yes<br>○ No<br>○ Unclear |

| | |
|---|---|
| or the point of diagnosis? e.g. "On average, patients had 5 encounters between initial provider contact/diagnosis to receiving treatment" | |
| Time to treatment<br>Do they give how long it took for patients to start treatment in time increments (e.g. precise dates or time in days, weeks, months, etc.)? | ○ Yes<br>○ No<br>○ Unclear |
| Location/provider where treatment was made<br>Does the paper say the type of health facility or provider the patients first received and continued their treatment at? | ○ Yes<br>○ No<br>○ Unclear |
| Proportions of patients per number of encounters to treatment<br>For each number of encounters, do the authors report the proportion of patients who had that many encounters between initial healthcare-seeking and treatment? Does the paper specify proportion per encounter for each step in the cascade up to treatment (First encounter after symptom onset -> Testing -> Diagnosis -> Treatment)? e.g. "20% of patients had 1-2 encounters, 30% had 3 encounters…" | ○ Yes<br>○ No<br>○ Unclear |
| Alignment of services<br>Do the authors do an analysis of the services available to the study population? (e.g. X% of clinics provide TB diagnosis, provide chest X-rays, provide Xpert) | ○ Yes<br>○ No<br>○ Unclear |

**Journey methodology & analysis**

| | |
|---|---|
| Name or description of journey methodology (type of chart)<br>e.g. Hansen-Chin, flowchart, Sankey chart, etc. Indicate if author's own methodology or replication of previous one. If author's own, indicate main components. | [Open text field] |
| Summary of analysis<br>Description of statistical analysis | [Open text field] |
| Outcome variables or major themes | [Open text field] |
| Strengths<br>If authors tried to limit recall bias, indicate how. | [Open text field] |
| Limitations | [Open text field] |
| Variables of interest or minor themes<br>Indicate the specific outcome that the variable is in reference to (cost, delay, access, etc.) | [Open text field] |

**Outcome measures**

| | |
|---|---|
| Delays<br>Select all that apply | □ Patient delay<br>□ Provider delay<br>□ Treatment delay<br>□ Diagnostic delay<br>□ Health system delay<br>□ Total delay<br>□ Delay, unspecified |

| | |
|---|---|
| | □ Other: __________________ |
| Journeys<br>Select all that apply | □ Number of visits to diagnosis<br>□ Number of visits to treatment<br>□ Type of provider for every visit<br>□ Sector of facility for every visit<br>□ Type of provider for place of initial healthcare-seeking<br>□ Sector of facility for place of initial healthcare-seeking<br>□ Type of provider for place of diagnosis<br>□ Sector of facility for place of diagnosis<br>□ Type of provider for place of treatment<br>□ Sector of facility for place of treatment<br>□ Other: _______________ |
| Other Methodology Variables<br>Select all that apply | □ Coverage of microscopy services among health facilities<br>□ Access of microscopy at initial healthcare-seeking<br>□ Coverage of treatment services among health facilities<br>□ Access of treatment at initial healthcare-seeking<br>□ Notification location<br>□ Treatment outcome |
| Time and date<br>Select all that apply | □ Time to initial healthcare-seeking<br>□ Time to diagnosis<br>□ Time to treatment<br>□ Date of first symptom<br>□ Date of initial care-seeking<br>□ Date of diagnosis<br>□ Date of referral for treatment<br>□ Date of treatment initiation<br>□ Date of treatment completion<br>□ Survival analysis (time to event/death)<br>□ Other: ____________ |
| Costs of care (at any point in care)<br>Select one | ○ Yes<br>○ No<br>○ Unclear |
| Other outcomes/major themes<br>Select one | ○ Yes<br>○ No<br>○ Unclear |
| Other, describe | [Open text field] |

**Other variables**

| | |
|---|---|
| <u>Patient characteristics</u><br>Select all that apply | □ Age<br>□ Sex/Gender<br>□ Race/Ethnicity<br>□ Education level<br>□ Literacy level<br>□ Place of residence<br>□ Urbanicity<br>□ Income level<br>□ Occupation<br>□ Number of persons/children in household<br>□ Socioeconomic status measure (e.g. material deprivation<br>□ Distance from nearest health facility<br>□ Marital status<br>□ Smoking status<br>□ Alcohol use<br>□ Other drug use<br>□ Comorbidities (any)<br>□ Type of illness<br>□ Severity of illness<br>□ HIV status<br>□ Diabetes status<br>□ Cause of death<br>□ Other: ___________________ |
| <u>Provider characteristics</u><br>Select all that apply | □ Age<br>□ Sex/Gender<br>□ Provider type<br>□ Sector of facility<br>□ Urbanicity<br>□ Other: ___________________ |
| <u>Healthcare/health system/healthcare-seeking characteristics</u><br>Select all that apply | □ Costs of care<br>□ Delay in accessing care<br>□ Number of visits<br>□ Type of provider of all instances of healthcare-seeking<br>□ Sector of facility of all instances of healthcare-seeking<br>□ Type of provider of initial care-seeking<br>□ Sector of facility of initial care-seeking<br>□ Type of provider of diagnosis<br>□ Sector of facility of diagnosis<br>□ Type of provider of treatment location |

| | |
|---|---|
| | □ Sector of facility of treatment location<br>□ Availability of services<br>□ Region/Sub-national area<br>□ Level of health facility<br>□ Other: _________________ |
| If qualitative, major/minor themes | [Open text field] |
