# Supplementary material for: A methodological review of patient healthcare-seeking journeys from symptom onset to receipt of care
Source: BMJ Glob Health. 2025 May 16;10(5):e016978. doi: 10.1136/bmjgh-2024-016978 (PMC12086929; doi:10.1136/bmjgh-2024-016978)
Supplement: online supplemental file 3 [file bmjgh-10-5-s003.pdf]

Supplement 3 – Quality assessment electronic forms

General information

**Reviewer Initials**	[Open text field]
**Study ID**	[Open text field]
**Study Title**	[Open text field]
**First author last name**	[Open text field]
**Year of publication**	[Open text field]
**Type of Study**<br>Select one	○ Qualitative Research<br>○ Mixed Methods<br>○ Analytical Cross-Sectional Study<br>○ Cohort Study<br>○ Prevalence Study

Qualitative Research Quality Assessment
Source: https://jbi.global/sites/default/files/2021-10/Checklist_for_Qualitative_Research.docx
Discussion of critical appraisal criteria are added before each question for context.

**1. Is there congruity between the stated philosophical perspective and the research methodology**<br><br>Does the report clearly state the philosophical or theoretical premises on which the study is based? Does the report clearly state the methodological approach adopted on which the study is based? Is there congruence between the two? For example:<br><br>A report may state that the study adopted a critical perspective and participatory action research methodology was followed. Here there is congruence between a critical view (focusing on knowledge arising out of critique, action and reflection) and action research (an approach that focuses on firstly working with groups to reflect on issues or practices, then considering how they could be different; then acting to create a change; and finally identifying new knowledge arising out of the action taken). However, a report may state that the study adopted an interpretive perspective and used survey methodology. Here there is incongruence between an interpretive view (focusing on knowledge arising out of studying what phenomena mean to individuals or groups) and surveys (an approach that focuses on asking standard questions to a defined study population); a report may state that the study was qualitative or used qualitative methodology (such statements do not demonstrate rigour in design) or make no statement on philosophical orientation or methodology.	○ Yes<br>○ No<br>○ Unclear<br>○ Not applicable

**2. Is there congruity between the research methodology and the research question or objectives?**<br>Is the study methodology appropriate for addressing the research question? For example:<br><br>A report may state that the research question was to seek understandings of the meaning of pain in a group of people with rheumatoid arthritis and that a phenomenological approach was taken. Here, there is congruity between this question and the methodology. A report may state that the research question was to establish the effects of counselling on the severity of pain experience and that an ethnographic approach was pursued. A question that tries to establish cause-and effect cannot be addressed by using an ethnographic approach (as ethnography sets out to develop understandings of cultural practices) and thus, this would be incongruent.	○ Yes<br>○ No<br>○ Unclear<br>○ Not applicable
**3. Is there congruity between the research methodology and the methods used to collect data**<br>Are the data collection methods appropriate to the methodology? For example:<br><br>A report may state that the study pursued a phenomenological approach and data was collected through phenomenological interviews. There is congruence between the methodology and data collection; a report may state that the study pursued a phenomenological approach and data was collected through a postal questionnaire. There is incongruence between the methodology and data collection here as phenomenology seeks to elicit rich descriptions of the experience of a phenomena that cannot be achieved through seeking written responses to standardized questions.	○ Yes<br>○ No<br>○ Unclear<br>○ Not applicable
**4. Is there congruity between the research methodology and the representation and analysis of data?**<br>Are the data analyzed and represented in ways that are congruent with the stated methodological position? For example:<br><br>A report may state that the study pursued a phenomenological approach to explore people's experience of grief by asking participants to describe their experiences of grief. If the text generated from asking these questions is searched to establish the meaning of grief to participants, and the meanings of all participants are included in the report findings, then this represents congruity; the same report may, however, focus only on those meanings that were	○ Yes<br>○ No<br>○ Unclear<br>○ Not applicable

common to all participants and discard single reported meanings. This would not be appropriate in phenomenological work.	
**5. Is there congruence between the research methodology and the interpretation of results?**<br>Are the results interpreted in ways that are appropriate to the methodology? For example:<br><br>A report may state that the study pursued a phenomenological approach to explore people's experience of facial disfigurement and the results are used to inform practitioners about accommodating individual differences in care. There is congruence between the methodology and this approach to interpretation; a report may state that the study pursued a phenomenological approach to explore people's experience of facial disfigurement and the results are used to generate practice checklists for assessment. There is incongruence between the methodology and this approach to interpretation as phenomenology seeks to understand the meaning of a phenomenon for the study participants and cannot be interpreted to suggest that this can be generalized to total populations to a degree where standardized assessments will have relevance across a population.	○ Yes<br>○ No<br>○ Unclear<br>○ Not applicable
**6. Is there a statement locating the researcher culturally or theoretically?**<br>Are the beliefs and values, and their potential influence on the study declared? For example:<br><br>The researcher plays a substantial role in the qualitative research process and it is important, in appraising evidence that is generated in this way, to know the researcher's cultural and theoretical orientation. A high quality report will include a statement that clarifies this.	○ Yes<br>○ No<br>○ Unclear<br>○ Not applicable
**7. Is the influence of the researcher on the research, and vice-versa, addressed?**<br>Is the potential for the researcher to influence the study and for the potential of the research process itself to influence the researcher and her/his interpretations acknowledged and addressed? For example:<br><br>Is the relationship between the researcher and the study participants addressed? Does the researcher critically examine her/his own role and potential influence during data collection? Is it reported how the researcher responded to events that arose during the study?	○ Yes<br>○ No<br>○ Unclear<br>○ Not applicable
**8. Are participants, and their voices, adequately represented?**<br>Generally, reports should provide illustrations from the data to show the basis of their conclusions and to ensure that participants are represented in the report.	○ Yes<br>○ No<br>○ Unclear<br>○ Not applicable

**9. Is the research ethical according to current criteria or, for recent studies, and is there evidence of ethical approval by an appropriate body?**<br>A statement on the ethical approval process followed should be in the report.	○ Yes<br>○ No<br>○ Unclear<br>○ Not applicable
**10. Do the conclusions drawn in the research report flow from the analysis, or interpretation, of the data?**<br>This criterion concerns the relationship between the findings reported and the views or words of study participants. In appraising a paper, appraisers seek to satisfy themselves that the conclusions drawn by the research are based on the data collected; data being the text generated through observation, interviews or other processes.	○ Yes<br>○ No<br>○ Unclear<br>○ Not applicable
**Overall appraisal**	○ Low quality<br>○ Medium quality<br>○ High quality<br>○ Seek further info
**Comments**	[Open text field]

Mixed Methods Quality Assessment

Adapted from the Mixed Methods Appraisal Tool. Citation: Hong QN, Pluye P, Fàbregues S, Bartlett G, Boardman F, Cargo M, Dagenais P, Gagnon M-P, Griffiths F, Nicolau B, O'Cathain A, Rousseau M-C, Vedel I. Mixed Methods Appraisal Tool (MMAT), version 2018. Registration of Copyright (#1148552), Canadian Intellectual Property Office, Industry Canada.

1. Are there clear research questions?	○ Yes<br>○ No<br>○ Unclear<br>○ Not applicable
2. Do the collected data allow the research questions to be addressed?	○ Yes<br>○ No<br>○ Unclear<br>○ Not applicable
**Part 1: Assessment of the Qualitative Aspects of the Study**	
Is the qualitative approach appropriate to answer the research question?	○ Yes<br>○ No<br>○ Unclear<br>○ Not applicable
Are the qualitative data collection methods adequate to address the research question?	○ Yes<br>○ No<br>○ Unclear<br>○ Not applicable
Are the findings adequately derived from the data?	○ Yes<br>○ No<br>○ Unclear<br>○ Not applicable

Is the interpretation of results sufficiently substantiated by data?	○ Yes<br>○ No<br>○ Unclear<br>○ Not applicable
Is there coherence between qualitative data sources, collection, analysis, and interpretation?	○ Yes<br>○ No<br>○ Unclear<br>○ Not applicable
**Part 2: Assessment of the Quantitative Aspects of the Study**	
Is the sampling strategy relevant to address the research question?	○ Yes<br>○ No<br>○ Unclear<br>○ Not applicable
Is the sample representative of the target population?	○ Yes<br>○ No<br>○ Unclear<br>○ Not applicable
Are the measurements appropriate?	○ Yes<br>○ No<br>○ Unclear<br>○ Not applicable
Is the risk of nonresponse bias low?	○ Yes<br>○ No<br>○ Unclear<br>○ Not applicable
**Is the statistical analysis appropriate to answer the research question?**	○ Yes<br>○ No<br>○ Unclear<br>○ Not applicable
**Part 3: Assessment of the Mixed Methods Aspects of the Study**	
Is there an adequate rationale for using a mixed methods design to address the research question?	○ Yes<br>○ No<br>○ Unclear<br>○ Not applicable
Are the different components of the study effectively integrated to answer the research question?	○ Yes<br>○ No<br>○ Unclear<br>○ Not applicable
Are the outputs of the integration of qualitative and quantitative components adequately interpreted?	○ Yes<br>○ No<br>○ Unclear<br>○ Not applicable
Are divergences and inconsistencies between quantitative and qualitative results adequately addressed?	○ Yes<br>○ No<br>○ Unclear<br>○ Not applicable

Do the different components of the study adhere to the quality criteria of each tradition of the methods involved?	○ Yes<br>○ No<br>○ Unclear<br>○ Not applicable
Overall appraisal	○ Low quality<br>○ Medium quality<br>○ High quality<br>○ Seek further info
Comments	[Open text field]

Analytical Cross-Sectional Quality Assessment
Source: https://jbi.global/sites/default/files/2021-10/Checklist_for_Analytical_Cross_Sectional_Studies.docx
Discussion of critical appraisal criteria are added before each question for context.

**1. Were the criteria for inclusion in the sample clearly defined?**<br>The authors should provide clear inclusion and exclusion criteria that they developed prior to recruitment of the study participants. The inclusion/exclusion criteria should be specified (e.g., risk, stage of disease progression) with sufficient detail and all the necessary information critical to the study.	○ Yes<br>○ No<br>○ Unclear<br>○ Not applicable
**2. Were the study subjects and the setting described in detail?**<br>The study sample should be described in sufficient detail so that other researchers can determine if it is comparable to the population of interest to them. The authors should provide a clear description of the population from which the study participants were selected or recruited, including demographics, location, and time period.	○ Yes<br>○ No<br>○ Unclear<br>○ Not applicable
**3. Was the exposure measured in a valid and reliable way?**<br>The study should clearly describe the method of measurement of exposure. Assessing validity requires that a 'gold standard' is available to which the measure can be compared. The validity of exposure measurement usually relates to whether a current measure is appropriate or whether a measure of past exposure is needed.<br><br>Reliability refers to the processes included in an epidemiological study to check repeatability of measurements of the exposures. These usually include intra-observer reliability and inter-observer reliability.	○ Yes<br>○ No<br>○ Unclear<br>○ Not applicable
**4. Were objective, standard criteria used for measurement of the condition?**<br>It is useful to determine if patients were included in the study based on either a specified diagnosis or definition. This is more likely to decrease the risk of bias. Characteristics are another useful approach to matching groups, and studies that did not use specified diagnostic methods or definitions should provide evidence on matching by key characteristics.	○ Yes<br>○ No<br>○ Unclear<br>○ Not applicable

**5. Were confounding factors identified?**<br>Confounding has occurred where the estimated intervention exposure effect is biased by the presence of some difference between the comparison groups (apart from the exposure investigated/of interest). Typical confounders include baseline characteristics, prognostic factors, or concomitant exposures (e.g. smoking). A confounder is a difference between the comparison groups and it influences the direction of the study results. A high quality study at the level of cohort design will identify the potential confounders and measure them (where possible). This is difficult for studies where behavioural, attitudinal or lifestyle factors may impact on the results.	○ Yes<br>○ No<br>○ Unclear<br>○ Not applicable
**6. Were strategies to deal with confounding factors stated?**<br>Strategies to deal with effects of confounding factors may be dealt within the study design or in data analysis. By matching or stratifying sampling of participants, effects of confounding factors can be adjusted for. When dealing with adjustment in data analysis, assess the statistics used in the study. Most will be some form of multivariate regression analysis to account for the confounding factors measured.	○ Yes<br>○ No<br>○ Unclear<br>○ Not applicable
**7. Were the outcomes measured in a valid and reliable way?**<br>Read the methods section of the paper. If for e.g. lung cancer is assessed based on existing definitions or diagnostic criteria, then the answer to this question is likely to be yes. If lung cancer is assessed using observer reported, or self-reported scales, the risk of over- or under-reporting is increased, and objectivity is compromised. Importantly, determine if the measurement tools used were validated instruments as this has a significant impact on outcome assessment validity.<br><br>Having established the objectivity of the outcome measurement (e.g. lung cancer) instrument, it's important to establish how the measurement was conducted. Were those involved in collecting data trained or educated in the use of the instrument/s? (e.g. radiographers). If there was more than one data collector, were they similar in terms of level of education, clinical or research experience, or level of responsibility in the piece of research being appraised?	○ Yes<br>○ No<br>○ Unclear<br>○ Not applicable
**8. Was appropriate statistical analysis used?**<br>As with any consideration of statistical analysis, consideration should be given to whether there was a more appropriate alternate statistical method that could have been used. The methods section should be detailed enough for reviewers to identify which analytical techniques were used (in particular, regression or stratification) and how specific confounders were measured.<br><br>For studies utilizing regression analysis, it is useful to identify if the study identified which variables were included and how they related	○ Yes<br>○ No<br>○ Unclear<br>○ Not applicable

to the outcome. If stratification was the analytical approach used, were the strata of analysis defined by the specified variables? Additionally, it is also important to assess the appropriateness of the analytical strategy in terms of the assumptions associated with the approach as differing methods of analysis are based on differing assumptions about the data and how it will respond.	
**Overall appraisal**	○ Low quality<br>○ Medium quality<br>○ High quality<br>○ Seek further info
**Comments**	[Open text field]

Cohort Study Quality Assessment
Source: https://jbi.global/sites/default/files/2021-10/Checklist_for_Cohort_Studies.docx
Discussion of critical appraisal criteria are added before each question for context.

**1. Were the two groups similar and recruited from the same population?**<br>Check the paper carefully for descriptions of participants to determine if patients within and across groups have similar characteristics in relation to exposure (e.g. risk factor under investigation). The two groups selected for comparison should be as similar as possible in all characteristics except for their exposure status, relevant to the study in question. The authors should provide clear inclusion and exclusion criteria that they developed prior to recruitment of the study participants.	○ Yes<br>○ No<br>○ Unclear<br>○ Not applicable
**2. Were the exposures measured similarly to assign people to both exposed and unexposed groups?**<br>A high-quality study at the level of cohort design should mention or describe how the exposures were measured. The exposure measures should be clearly defined and described in detail. This will enable reviewers to assess whether or not the participants received the exposure of interest.	○ Yes<br>○ No<br>○ Unclear<br>○ Not applicable
**3. Was the exposure measured in a valid and reliable way?**<br>The study should clearly describe the method of measurement of exposure. Assessing validity requires that a 'gold standard' is available to which the measure can be compared. The validity of exposure measurement usually relates to whether a current measure is appropriate or whether a measure of past exposure is needed.<br><br>Reliability refers to the processes included in an epidemiological study to check repeatability of measurements of the exposures. These usually include intra-observer reliability and inter-observer reliability.	○ Yes<br>○ No<br>○ Unclear<br>○ Not applicable
**4. Were confounding factors identified?**	○ Yes

Confounding has occurred where the estimated intervention exposure effect is biased by the presence of some difference between the comparison groups (apart from the exposure investigated/of interest). Typical confounders include baseline characteristics, prognostic factors, or concomitant exposures (e.g. smoking). A confounder is a difference between the comparison groups and it influences the direction of the study results. A high quality study at the level of cohort design will identify the potential confounders and measure them (where possible). This is difficult for studies where behavioral, attitudinal or lifestyle factors may impact on the results.	○ No<br>○ Unclear<br>○ Not applicable
**5. Were strategies to deal with confounding factors stated?**<br>Strategies to deal with effects of confounding factors may be dealt within the study design or in data analysis. By matching or stratifying sampling of participants, effects of confounding factors can be adjusted for. When dealing with adjustment in data analysis, assess the statistics used in the study. Most will be some form of multivariate regression analysis to account for the confounding factors measured. Look out for a description of statistical methods as regression methods such as logistic regression are usually employed to deal with confounding factors/variables of interest.	○ Yes<br>○ No<br>○ Unclear<br>○ Not applicable
**6. Were the groups/participants free of the outcome at the start of the study (or at the moment of exposure)?**<br>The participants should be free of the outcomes of interest at the start of the study. Refer to the 'methods' section in the paper for this information, which is usually found in descriptions of participant/sample recruitment, definitions of variables, and/or inclusion/exclusion criteria.	○ Yes<br>○ No<br>○ Unclear<br>○ Not applicable
**7. Were the outcomes measured in a valid and reliable way?**<br>Read the methods section of the paper. If for e.g. lung cancer is assessed based on existing definitions or diagnostic criteria, then the answer to this question is likely to be yes. If lung cancer is assessed using observer reported, or self-reported scales, the risk of over- or under-reporting is increased, and objectivity is compromised. Importantly, determine if the measurement tools used were validated instruments as this has a significant impact on outcome assessment validity.<br><br>Having established the objectivity of the outcome measurement (e.g. lung cancer) instrument, it's important to establish how the measurement was conducted. Were those involved in collecting data trained or educated in the use of the instrument/s? (e.g. radiographers). If there was more than one data collector, were they similar in terms of level of education, clinical or research experience, or level of responsibility in the piece of research being appraised?	○ Yes<br>○ No<br>○ Unclear<br>○ Not applicable
**8. Was the follow up time reported and sufficient to be long enough for outcomes to occur?**	○ Yes<br>○ No

The appropriate length of time for follow up will vary with the nature and characteristics of the population of interest and/or the intervention, disease or exposure. To estimate an appropriate duration of follow up, read across multiple papers and take note of the range for duration of follow up. The opinions of experts in clinical practice or clinical research may also assist in determining an appropriate duration of follow up. For example, a longer timeframe may be needed to examine the association between occupational exposure to asbestos and the risk of lung cancer. It is important, particularly in cohort studies that follow up is long enough to enable the outcomes. However, it should be remembered that the research question and outcomes being examined would probably dictate the follow up time.	○ Unclear<br>○ Not applicable
**9. Was follow up complete, and if not, were the reasons to loss to follow up described and explored?**<br>It is important in a cohort study that a greater percentage of people are followed up. As a general guideline, at least 80% of patients should be followed up. Generally a dropout rate of 5% or less is considered insignificant. A rate of 20% or greater is considered to significantly impact on the validity of the study. However, in observational studies conducted over a lengthy period of time a higher dropout rate is to be expected. A decision on whether to include or exclude a study because of a high dropout rate is a matter of judgement based on the reasons why people dropped out, and whether dropout rates were comparable in the exposed and unexposed groups.<br><br>Reporting of efforts to follow up participants that dropped out may be regarded as an indicator of a well conducted study. Look for clear and justifiable description of why people were left out, excluded, dropped out etc. If there is no clear description or a statement in this regards, this will be a 'No'.	○ Yes<br>○ No<br>○ Unclear<br>○ Not applicable
**10. Were strategies to address incomplete follow up utilized?**<br>Some people may withdraw due to change in employment or some may die; however, it is important that their outcomes are assessed. Selection bias may occur as a result of incomplete follow up. Therefore, participants with unequal follow up periods must be taken into account in the analysis, which should be adjusted to allow for differences in length of follow up periods. This is usually done by calculating rates which use person-years at risk, i.e. considering time in the denominator.	○ Yes<br>○ No<br>○ Unclear<br>○ Not applicable
**11. Was appropriate statistical analysis used?**<br>As with any consideration of statistical analysis, consideration should be given to whether there was a more appropriate alternate statistical method that could have been used. The methods section of cohort studies should be detailed enough for reviewers to identify which	○ Yes<br>○ No<br>○ Unclear<br>○ Not applicable

analytical techniques were used (in particular, regression or stratification) and how specific confounders were measured.<br><br>For studies utilizing regression analysis, it is useful to identify if the study identified which variables were included and how they related to the outcome. If stratification was the analytical approach used, were the strata of analysis defined by the specified variables? Additionally, it is also important to assess the appropriateness of the analytical strategy in terms of the assumptions associated with the approach as differing methods of analysis are based on differing assumptions about the data and how it will respond.	
**Overall appraisal**	○ Low quality<br>○ Medium quality<br>○ High quality<br>○ Seek further info
**Comments**	[Open text field]

Prevalence Study Quality Assessment
Source: https://jbi.global/sites/default/files/2021-10/Checklist_for_Prevalence_Studies.docx
Discussion of critical appraisal criteria are added before each question for context.

**1. Was the sample frame appropriate to address the target population?**<br>**This question relies upon knowledge of the broader characteristics of** the population of interest and the geographical area. If the study is of women with breast cancer, knowledge of at least the characteristics, demographics and medical history is needed. The term “target population” should not be taken to infer every individual from everywhere or with similar disease or exposure characteristics. Instead, give consideration to specific population characteristics in the study, including age range, gender, morbidities, medications, and other potentially influential factors. For example, a sample frame may not be appropriate to address the target population if a certain group has been used (such as those working for one organisation, or one profession) and the results then inferred to the target population (i.e. working adults). A sample frame may be appropriate when it includes almost all the members of the target population (i.e. a census, or a complete list of participants or complete registry data).	○ Yes<br>○ No<br>○ Unclear<br>○ Not applicable
**2. Were study participants recruited in an appropriate way?**<br>Studies may report random sampling from a population, and the methods section should report how sampling was performed. Random probabilistic sampling from a defined subset of the population (sample frame) should be employed in most cases, however, random probabilistic sampling is not needed when	○ Yes<br>○ No<br>○ Unclear<br>○ Not applicable

everyone in the sampling frame will be included/analysed. For example, reporting on all the data from a good census is appropriate as a good census will identify everybody. When using cluster sampling, such as a random sample of villages within a region, the methods need to be clearly stated as the precision of the final prevalence estimate incorporates the clustering effect. Convenience samples, such as a street survey or interviewing lots of people at a public gatherings are not considered to provide a representative sample of the base population.	
**3. Was the sample size adequate?**<br>The larger the sample, the narrower will be the confidence interval around the prevalence estimate, making the results more precise. An adequate sample size is important to ensure good precision of the final estimate. Ideally we are looking for evidence that the authors conducted a sample size calculation to determine an adequate sample size. This will estimate how many subjects are needed to produce a reliable estimate of the measure(s) of interest. For conditions with a low prevalence, a larger sample size is needed. Also consider sample sizes for subgroup (or characteristics) analyses, and whether these are appropriate. Sometimes, the study will be large enough (as in large national surveys) whereby a sample size calculation is not required. In these cases, sample size can be considered adequate. When there is no sample size calculation and it is not a large national survey, the reviewers may consider conducting their own sample size analysis.	○ Yes<br>○ No<br>○ Unclear<br>○ Not applicable
**4. Were the study subjects and setting described in detail?**<br>Certain diseases or conditions vary in prevalence across different geographic regions and populations (e.g. Women vs. Men, sociodemographic variables between countries). The study sample should be described in sufficient detail so that other researchers can determine if it is comparable to the population of interest to them.	○ Yes<br>○ No<br>○ Unclear<br>○ Not applicable
**5. Was data analysis conducted with sufficient coverage of the identified sample?**<br>Coverage bias can occur when not all subgroups of the identified sample respond at the same rate. For instance, you may have a very high response rate overall for your study, but the response rate for a certain subgroup (i.e. older adults) may be quite low.	○ Yes<br>○ No<br>○ Unclear<br>○ Not applicable
**6. Were valid methods used for the identification of the condition?**<br>Here we are looking for measurement or classification bias. Many health problems are not easily diagnosed or defined and some measures may not be capable of including or excluding appropriate levels or stages of the health problem. If the outcomes were assessed	○ Yes<br>○ No<br>○ Unclear<br>○ Not applicable

based on existing definitions or diagnostic criteria, then the answer to this question is likely to be yes. If the outcomes were assessed using observer reported, or self-reported scales, the risk of over- or under-reporting is increased, and objectivity is compromised. Importantly, determine if the measurement tools used were validated instruments as this has a significant impact on outcome assessment validity.	
**7. Was the condition measured in a standard, reliable way for all participants?**<br>Considerable judgment is required to determine the presence of some health outcomes. Having established the validity of the outcome measurement instrument (see item 6 of this scale), it is important to establish how the measurement was conducted. Were those involved in collecting data trained or educated in the use of the instrument/s? If there was more than one data collector, were they similar in terms of level of education, clinical or research experience, or level of responsibility in the piece of research being appraised? When there was more than one observer or collector, was there comparison of results from across the observers? Was the condition measured in the same way for all participants?	○ Yes<br>○ No<br>○ Unclear<br>○ Not applicable
**8. Was there appropriate statistical analysis?**<br>Importantly, the numerator and denominator should be clearly reported, and percentages should be given with confidence intervals. The methods section should be detailed enough for reviewers to identify the analytical technique used and how specific variables were measured. Additionally, it is also important to assess the appropriateness of the analytical strategy in terms of the assumptions associated with the approach as differing methods of analysis are based on differing assumptions about the data and how it will respond.	○ Yes<br>○ No<br>○ Unclear<br>○ Not applicable
**9. Was the response rate adequate, and if not, was the low response rate managed appropriately?**<br>A large number of dropouts, refusals or "not founds" amongst selected subjects may diminish a study's validity, as can a low response rates for survey studies. The authors should clearly discuss the response rate and any reasons for non-response and compare persons in the study to those not in the study, particularly with regards to their socio-demographic characteristics. If reasons for non-response appear to be unrelated to the outcome measured and the characteristics of non-responders are comparable to those who do respond in the study (addressed in question 5, coverage bias), the researchers may be able to justify a more modest response rate.	○ Yes<br>○ No<br>○ Unclear<br>○ Not applicable
**Overall appraisal**	○ Low quality

	○ Medium quality<br>○ High quality<br>○ Seek further info
**Comments**	[Open text field]
