# Supplementary material for: A methodological review of patient healthcare-seeking journeys from symptom onset to receipt of care
Source: BMJ Glob Health. 2025 May 16;10(5):e016978. doi: 10.1136/bmjgh-2024-016978 (PMC12086929; doi:10.1136/bmjgh-2024-016978)
Supplement: online supplemental file 4 [file bmjgh-10-5-s004.pdf]

Supplement 4 – Quality appraisal

S/No	Study Title	First author last name	Year of publication	Type of Study	Overall appraisal
1	Health care seeking behavior and patient delay in tuberculosis diagnosis	Almeida	2015	Analytical Cross-Sectional	High
2	The road to tuberculosis treatment in rural Nepal: A qualitative assessment of 26 journeys	Asbroek	2008	Qualitative	High
3	Delays to anti-tuberculosis treatment intiation among cases on directly observed treatment short course in districts of southwestern Ethiopia: a cross sectional study	Asres	2019	Analytical Cross-Sectional	High
4	Such a long journey: What health seeking pathways of patients with drug resistant tuberculosis in Mumbai tell us	Bhattacharya	2019	Qualitative	High
5	How do patients access the private sector in Chennai, India? An evaluation of delays in tuberculosis diagnosis	Bronner Murrison	2016	Cross-Sectional Study	High
6	Health-seeking behaviour and treatment delay in patients with pulmonary tuberculosis in Switzerland: some slip through the net	Christian	2018	Analytical Cross-Sectional	High
7	Delays in TB Diagnosis and Treatment Initiation in Burkina Faso during the COVID-19 Pandemic	Diallo	2022	Analytical Cross-Sectional	High
8	Health seeking behaviour and delayed management of tuberculosis patients in rural Bangladesh	Ehsanul Huq	2018	Analytical Cross-Sectional	High
9	Real-world colorectal cancer diagnostic pathways in Ontario, Canada: A population-based study	Guan	2022	Analytical Cross-Sectional	High
10	Patient pathways of tuberculosis care- seeking and treatment: an individual- level analysis of National Health Insurance data in Taiwan	Ku	2020	Cohort	High
11	Health-seeking behaviour for febrile illness in malaria-endemic Kolasib district, Mizoram, India	Lalchhuanawma	2012	Analytical Cross-Sectional	High
12	Patient pathways and delays to diagnosis and treatment of tuberculosis in an urban setting in Indonesia	Lestari	2020	Analytical Cross-Sectional	High

S/No	Study Title	First author last name	Year of publication	Type of Study	Overall appraisal
13	Health-seeking pathway and factors leading to delays in tuberculosis diagnosis in West Pokot County, Kenya: a grounded theory study	Mbuthia	2018	Qualitative	High
14	Delayed consultation among pulmonary tuberculosis patients: a cross sectional study of 10 DOTS districts of Ethiopia	Mesfin	2009	Analytical Cross-Sectional	High
15	Patterns and predictors of malaria care-seeking, diagnostic testing, and artemisinin-based combination therapy for children under five with fever in Northern Nigeria: a cross-sectional study	Millar	2014	Analytical Cross-Sectional	High
16	Demand for malaria rapid diagnostic test, health care-seeking behaviour, and drug use among rural community members with fever or malaria-like illness in Ebonyi state, Nigeria: a cross-sectional household survey	Omale	2021	Analytical Cross-Sectional	High
17	Care-seeking during fatal childhood illness in rural South Africa: a qualitative study	Price	2021	Qualitative	High
18	Effect of public-private interface agency in Patna and Mumbai, India: Does it alter durations and delays in care seeking for drug-sensitive pulmonary tuberculosis?	Shah	2020	Analytical Cross-Sectional	High
19	Healthcare-seeking behavior, treatment delays and its determinants among pulmonary tuberculosis patients in rural Nigeria: a cross-sectional study	Ukwaja	2013	Analytical Cross-Sectional	High
20	Tuberculosis patients' knowledge and beliefs about tuberculosis: a mixed methods study from the Pacific Island nation of Vanuatu	Viney	2014	Mixed Methods	High
21	Comparing patient care seeking pathways in three models of hospital and TB programme collaboration in China	Wei	2013	Analytical Cross-Sectional	High
22	Transaction costs of access to health care: Implications of the care-seeking pathways of tuberculosis patients for health system governance in Nigeria	Abimbola	2015	Cross-Sectional Study	Medium

S/No	Study Title	First author last name	Year of publication	Type of Study	Overall appraisal
23	Diagnostic delay amongst tuberculosis patients in Jogjakarta Province, Indonesia is related to the quality of services in DOTS facilities	Ahmad	2011	Cross-Sectional Study	Medium
24	Knowledge on the transmission, prevention and treatment of malaria among two endemic populations of Bangladesh and their health-seeking behaviour	Ahmed	2009	Cross-Sectional Study	Medium
25	Duration of intervals in the care seeking pathway for lung cancer in Bangladesh: A journey from symptoms triggering consultation to receipt of treatment	Ansar	2021	Cross-Sectional Study	Medium
26	Patient's site of first access to health system influences length of delay for tuberculosis treatment in Tajikistan	Aye	2010	Cross-Sectional Study	Medium
27	Care seeking and attitudes towards treatment compliance by newly enrolled tuberculosis patients in the district treatment programme in rural western Kenya: a qualitative study	Ayisi	2011	Qualitative Research	Medium
28	Men with sexually transmitted diseases in Bangkok: where do they go for treatment and why?	Benjarattanaporn	1997	Cross-Sectional Study	Medium
29	Pre-treatment delay and out of pocket expenses by notified new tuberculosis patients in an Indian mega city	Chadha	2022	Cross-Sectional Study	Medium
30	Care Seeking Behavior of Chest Symptomatics: A Community Based Study Done in South India after the Implementation of the RNTCP	Charles	2010	Cross-Sectional Study	Medium
31	Cost of seeking care for tuberculosis since the implementation of universal health coverage in Indonesia	Fuady	2020	Cross-Sectional Study	Medium
32	Care seeking behaviour and various delays in tuberculosis patients registered under RNTCP in Pune city	Gothankar	2016	Cross-Sectional Study	Medium

S/No	Study Title	First author last name	Year of publication	Type of Study	Overall appraisal
33	The relationship between poverty and healthcare seeking among patients hospitalized with acute febrile illnesses in Chittagong, Bangladesh	Herdman	2016	Cross-Sectional Study	Medium
34	Healthcare Seeking and Access to Care for Pneumonia, Sepsis, Meningitis, and Malaria in Rural Gambia	Hossain	2022	Cross-Sectional Study	Medium
35	Barriers on the pathway to survival for children dying from treatable illnesses in Inhambane province, Mozambique	Kallander	2019	Cross-Sectional Study	Medium
36	Study of factors leading to treatment delay in new sputum positive pulmonary tuberculosis patients and its impact on sputum conversion	Kumawat	2022	Cohort Study	Medium
37	Patients are paying too much for tuberculosis: a direct cost-burden evaluation in Burkina Faso	Laokri	2013	Cross-Sectional Study	Medium
38	Case-finding tuberculosis patients: diagnostic and treatment delays and their determinants	Maamari	2008	Cross-Sectional Study	Medium
39	How much do delayed healthcare seeking, delayed care provision, and diversion from primary care contribute to the transmission of STIs?	Mercer	2007	Cross-Sectional Study	Medium
40	Pulmonary tuberculosis in Patna, India: Durations, delays, and health care seeking behaviour among patients identified through household surveys	Mistry	2017	Cross-Sectional Study	Medium
41	Durations and Delays in Care Seeking, Diagnosis and Treatment Initiation in Uncomplicated Pulmonary Tuberculosis Patients in Mumbai, India	Mistry	2016	Mixed Methods	Medium
42	Patient navigation pathway and barriers to treatment seeking in cancer in India: A qualitative inquiry	Pati	2013	Mixed Methods	Medium
43	Care-seeking pathways, care challenges, and coping experiences of rural women living with rheumatoid arthritis in Odisha, India	Pati	2019	Qualitative Research	Medium
44	Factors associated with patient and health system delays in the diagnosis of tuberculosis in South India	Rajeswari	2002	Cross-Sectional Study	Medium

S/No	Study Title	First author last name	Year of publication	Type of Study	Overall appraisal
45	Patient treatment pathways of multidrug-resistant tuberculosis cases in coastal South India: Road to a drug resistant tuberculosis center	Rathi	2021	Cross-Sectional Study	Medium
46	Care seeking behaviour and diagnostic processes in patients with smear-positive pulmonary tuberculosis in Malawi	Salaniponi	2000	Cross-Sectional Study	Medium
47	A comparison of patient treatment pathways among multidrug-resistant and drug-sensitive TB cases in Delhi, India: A cross-sectional study	Sharma	2020	Cross-Sectional Study	Medium
48	From fever to anti-malarial: the treatment-seeking process in rural Senegal	Smith	2010	Cross-Sectional Study	Medium
49	Pathways to the first contact with specialist mental health care	Steel	2006	Cross-Sectional Study	Medium
50	Health care seeking patterns of rifampicin-resistant tuberculosis patients in Harare, Zimbabwe: A prospective cohort study	Tadokera	2021	Mixed Methods	Medium
51	Understanding health care-seeking behaviour of the tribal population in India among those with presumptive TB symptoms	Thomas	2021	Cross-Sectional Study	Medium
52	Diagnostic pathways and direct medical costs incurred by new adult pulmonary tuberculosis patients prior to anti-tuberculosis treatment â€“ Tamil Nadu, India	Veesa	2018	Cross-Sectional Study	Medium
53	Analysis of care-seeking pathways of tuberculosis patients in Guangxi, China, with and without decentralised tuberculosis services	Wei	2009	Cross-Sectional Study	Medium
54	Patient care pathways under the model of integrating tuberculosis service with general hospitals in China	Wei	2013	Cross-Sectional Study	Medium
55	The pathway to diagnosis and treatment for surgically managed lung cancer patients	White	2020	Cross-Sectional Study	Medium
56	Healthcare utilization for common infectious disease syndromes in Soweto and Klerksdorp, South Africa	Wong	2018	Cross-Sectional Study	Medium

S/No	Study Title	First author last name	Year of publication	Type of Study	Overall appraisal
57	Patients pathways to tuberculosis diagnosis and treatment in a fragmented health system: a qualitative study from a south Indian district	Yellappa	2017	Qualitative Research	Medium
58	Patient pathway analysis of tuberculosis diagnostic delay: a multicentre retrospective cohort study in China	Zhang	2021	Cohort Study	Medium
59	Care Seeking Behavior of the TB Patients who were Registered in an Urban Government Tuberculosis Control in Chennai, Tamilnadu, India	Ananthakrishnan	2012	Cross-Sectional Study	Low
60	Tuberculosis pathways to care and transmission of multidrug resistance in India	Atre	2022	Mixed Methods	Low
61	Health seeking behaviour, health system experience and tuberculosis case finding in Gambians with cough	Kasse	2006	Cross-Sectional Study	Low
62	Idiopathic pulmonary fibrosis: Physician and patient perspectives on the pathway to care from symptom recognition to diagnosis and disease burden	Lancaster	2021	Cross-Sectional Study	Low
63	Pathways and costs of care for patients with tuberculosis symptoms in rural Uganda	Shete	2015	Cross-Sectional Study	Low
64	Health seeking and knowledge about tuberculosis among persons with pulmonary symptoms and tuberculosis cases in Bangalore slums	Suganthi	2008	Cross-Sectional Study	Low
65	Health-seeking behavior of COVID-19 cases during the first eight weeks of the outbreak in Singapore: differences between local community and imported cases and having visits to single or multiple healthcare providers	Tay	2022	Cross-Sectional Study	Low
66	A Cross-Sectional Descriptive Study to Ascertain Factors Influencing Delay in Diagnosis among Newly Diagnosed Pulmonary Tuberculosis Patients	Thomas	2022	Cross-Sectional Study	Low
